# Recent Advances in Nanotechnology Therapeutics

**DOI:** 10.3390/pharmaceutics18070891

**Published:** 2026-07-20

**Authors:** Hyun-Ouk Kim

**Affiliations:** 1 Division of Chemical Engineering and Bioengineering, College of Art, Culture and Engineering, Kangwon National University, Chuncheon-si 24341, Gangwon-do, Republic of Korea; kimhoman@kangwon.ac.kr; 2 Department of Smart Health Science and Technology, Kangwon National University, Chuncheon-si 24341, Gangwon-do, Republic of Korea; 3 Institute of Fermentation of Brewing, Kangwon National University, Chuncheon-si 24341, Gangwon-do, Republic of Korea

## 1. Introduction

Nanotechnology has moved from a promising idea to a working part of how we treat disease. Over the past two decades, the ability to design particles at the nanometer scale has given researchers a way to carry drugs to places that were once hard to reach, to protect fragile payloads such as nucleic acids, and to formulate treatments that would otherwise be difficult to deliver. What began largely in oncology has since spread into gene editing, wound healing, cardiovascular disease, and antiviral therapy.

This Special Issue of *Pharmaceutics*, Recent Advances in Nanotechnology Therapeutics, takes a wide view of that expansion and gathers work on delivery, formulation, and treatment across a range of conditions. It gathered eleven contributions from groups working on several continents, and this editorial offers a short tour through the study directions they represent.

## 2. Overview of the Contributions

The contributions show how far nanotherapeutics now reach beyond cancer. Gene delivery is a strong thread. Kim and colleagues reviewed nanoparticles as non-viral vectors for CRISPR/Cas9, weighing them against the viral systems that have long dominated the field (Contribution 4). Neves and co-workers took a more mechanistic route, showing that a peptide-drug and p53 gene complex can promote cognate gene expression and reduce the viability of glioblastoma cells (Contribution 3).

Formulation and manufacturing questions, which often decide whether a nanomedicine can be scaled, are addressed directly. Chiesa and colleagues examined how micromixer geometry affects the manufacturing of lipid nanocarriers for proteins and nucleic acids, a practical study for anyone moving from bench to production (Contribution 1). Lee and co-workers developed C24 ceramide lipid nanoparticles for skin wound healing, extending nanoparticle formulation into regenerative medicine (Contribution 6). The issue also reaches into cardiovascular disease, with two contributions on atherosclerosis. Lin and colleagues reviewed photodynamic therapy as a minimally invasive option (Contribution 2), while Cui and co-workers described cyclodextrin acting as both a therapeutic agent and a nanocarrier for regulating cholesterol homeostasis (Contribution 11).

Several other works fill out the therapeutic range of this issue. These include resveratrol-loaded nanoparticles tested against lung tumor spheroids (Contribution 5), silver dimolybdate nanorods with anticancer activity against breast and prostate tumors (Contribution 7), nanotechnology-based strategies for preventing post-surgical adhesions (Contribution 8), an andiroba oil-based nanoemulsion with in vitro healing potential (Contribution 9), and a screening-identified oxazole-4-carboxamide lead with in vitro anti-coronavirus activity (Contribution 10). The breadth here is the point. The same design principles that serve oncology are being adapted to wound care, cardiovascular disease, and infectious disease.

## 3. Outlook and Future Directions

Reading these eleven contributions together, a few gaps stand out as the natural focus for future study. Manufacturing reproducibility is a recurring theme, and the study on micromixer design is a reminder that elegant particles are only useful if they can be made the same way at scale. Delivery specificity is a second, whether the target is an atherosclerotic plaque, a wound bed, or a solid tumor. Biocompatibility and long-term safety remain a third, relevant to nearly every platform here. A fourth question, harder to answer, is how well results from in vitro spheroids and animal models will hold up in patients.

The direction of travel seems clear. Gene-editing payloads, mRNA therapeutics, and combination approaches that pair delivery with immune activation are likely to draw the most attention in the coming years, and non-viral delivery is central to all three. Progress will depend as much on careful formulation science and honest safety assessment as on new materials. It is my hope that this Reprint gives readers both a snapshot of where nanotherapeutics stand and a sense of the questions worth pursuing next.

